# Supplementary material for: Semiparametric regression and risk prediction with competing risks data under missing cause of failure
Source: Lifetime Data Anal. 2020 Jan 25;26(4):659–84. doi: 10.1007/s10985-020-09494-1 (PMC7381366; doi:10.1007/s10985-020-09494-1)
Supplement: Supplementary file 1 — Supplementary material 1 (pdf 163 KB) [file 10985_2020_9494_MOESM1_ESM.pdf]

Noname manuscript No.
(will be inserted by the editor)

Electronic Supplementary Material

Semiparametric regression and risk prediction with competing risks data under missing cause of failure

Received: date / Accepted: date

Abstract This Electronic Supplementary Material contains R code for the implementation of the proposed methodology, proofs of the theorems presented in Section 3.2 of the main text, explicit formulas for the estimated influence functions, and simulation study results in terms of the infinite-dimensional parameters.

PACS 02.50.r

Mathematics Subject Classification (2010) 62N01 · 62N02

1 R Code

Our estimation approach can be easily applied using the `coxph` function of the R package `survival`. Here we illustrate the use of `coxph` with missing event type according to our methodology. First, consider a dataset named `data` with competing risks, which includes failure time `x`, event type `c`, a covariate of interest `z`, and an auxilliary covariate `a`. The first step of the analysis is to fit a logistic model for the probability of the cause of interest. First define a event type, i.e.

```
cause <- 1.
```

Next, one needs to fit the logistic model $\pi_j(\mathbf{W}_i, \boldsymbol{\gamma}_0)$ in the complete cases and then calculate the "weight"

$$R_i \Delta_{ij} + (1 - R_i)\pi_j(\mathbf{W}_i, \hat{\boldsymbol{\gamma}}_n).$$

This can be done using the following lines of code:

```
data[data$r==0,"c"]<- -1
cc <- data[data$r==1 & data$c>0,]
cc$y <- 1*(cc$c==cause)
```

```
mod1 <- glm(y ~ x + z + a, family = "binomial", data = cc)
data$pi <- predict(mod1, data, type = "response")
data$weight <- data$r*(data$c==cause)+(1-data$r)*data$pi
data$d <- (data$weight>0)
data$weight <- data$weight + (data$weight == 0)
```

The next stage of the analysis is to fit the Cox proportional hazards model using `data$weight` as weight in the `coxph` function. However, since case weights in `coxph` are also incuded in the risk sets, we need a data manipulation step in order to "remove" these weights from the risk sets for the observations with a missing event type. This data manipulation step proceeds as follows:

```
dt0 <- data[data$r==0,]
dt0$weight <- 1 - dt0$weight
dt0$d <- 0
data1 <- rbind(data,dt0)
```

Now estimation of the regression coefficients using the proposed maximum pseudo partial likelihood approach is performed by using `coxph` in the augmented dataset `data1`, with the weights `data1$weight`:

```
fit <- coxph(Surv(x, d) ~ z, weight = weight, data = data1)
b1 <- coef(fit)
```

Note that the standard error estimates provided by `coxph` are not valid and correct estimation of the standard errors can be performed using the nonparametric bootstrap. We plan to develop an R package that provides standard errors according to our closed-form estimators.

Estimation of the baseline cumulative cause-specific hazard functions can be performed using the `basehaz` function as:

```
H1 <- basehaz(fit, centered = FALSE)
```

Finally, the cumulative incidence function given the covariate pattern $\mathbf{z}_0$ can be easily estimated using the regression coefficient `b1` and baseline cumulative cause-specific hazard estimates for all cases of failure using the estimator:

$$\hat{F}_{n,1}(t;\mathbf{z}_0) = \int_0^t \exp\left[-\sum_{l=1}^{k} \hat{\Lambda}_{n,l}(s-;\mathbf{z}_0)\right] d\hat{\Lambda}_{n,1}(s;\mathbf{z}_0), \qquad t \in [0,\tau].$$

For example, with two possible causes of failure, the baseline cumulative incidence function for cause 1 can be estimated based on the baseline cumulative cause-specific hazards `H1` and `H2` as follows:

```
Haz1 <- H1$hazard
Haz2 <- H2$hazard
S <- exp(-Haz1-Haz2)
S.minus <- c(1,S[1:(length(S)-1)])
Haz1.minus <- c(0,Haz1[1:(length(Haz1)-1)])
CIF1 <- cumsum(S.minus*(Haz1-Haz1.minus))
```

Standard error estimation for the covariate-specific cumulative incidence function can again be performed by using the nonparametric bootstrap method. As noted above, we plan to develop an R package that implements the proposed methods with full functionality.

2 Asymptotic Theory Proofs

Our study of the asymptotic properties of the proposed estimators heavily rely on empirical process theory [4,2]. We use the standard notation

$$\mathbb{P}_n f = \frac{1}{n}\sum_{i=1}^{n} f(\mathbf{D}_i), \text{ and } Pf = \int_{\mathcal{D}} f dP = Ef,$$

for any measurable function $f : \mathcal{D} \mapsto \mathbb{R}$, where $\mathcal{D}$ denotes the sample space and P the true (induced) probability measure defined on the Borel σ-algebra on $\mathcal{D}$. Let K be a generic constant that my differ from place to place. In many calculations we only focus on one arbitrarily chosen event type j, as the same technical proofs apply to all $j = 1, \ldots, k$.

2.1 Proof of Theorem 1

To facilitate the presentation of the proofs we define

$$\mathbf{G}_j(\boldsymbol{\beta}_j) = P\left\{\int_0^\tau \left[\mathbf{Z} - E(t, \boldsymbol{\beta}_j)\right] d\tilde{N}_j(t; \boldsymbol{\gamma}_0)\right\}$$

for $j = 1, \ldots, k$. Note that $\mathbf{G}_{n,j}(\boldsymbol{\beta}_j; \hat{\boldsymbol{\gamma}}_n)$ defined in Section 3.1 can be rewritten as

$$\mathbf{G}_{n,j}(\boldsymbol{\beta}_j; \hat{\boldsymbol{\gamma}}_n) = \mathbb{P}_n\left\{\int_0^\tau [\mathbf{Z} - E_n(t, \boldsymbol{\beta}_j)] d\tilde{N}_j(t; \hat{\boldsymbol{\gamma}}_n)\right\}$$

Trivial algebra leads to the decomposition

$$\mathbf{G}_{n,j}(\boldsymbol{\beta}_j; \hat{\boldsymbol{\gamma}}_n) - \mathbf{G}_j(\boldsymbol{\beta}_j) = \mathbf{A}_{n,j} + \mathbf{B}_{n,j} - \mathbf{C}_{n,j}(\boldsymbol{\beta}_j) - \mathbf{D}_{n,j}(\boldsymbol{\beta}_j), \quad j = 1, \ldots, k,$$

where

$$\mathbf{A}_{n,j} = \mathbb{P}_n\left\{\int_0^\tau \mathbf{Z}\left[d\tilde{N}_j(t; \hat{\boldsymbol{\gamma}}_n) - d\tilde{N}_j(t; \boldsymbol{\gamma}_0)\right]\right\},$$

$$\mathbf{B}_{n,j} = (\mathbb{P}_n - P)\int_0^\tau \mathbf{Z} d\tilde{N}_j(t; \boldsymbol{\gamma}_0),$$

$$\mathbf{C}_{n,j}(\boldsymbol{\beta}_j) = \mathbb{P}_n\left\{\int_0^\tau \left[E_n(t, \boldsymbol{\beta}_j) - E(t; \boldsymbol{\beta}_j)\right] d\tilde{N}_j(t; \hat{\boldsymbol{\gamma}}_n)\right\}$$

and

$$\mathbf{D}_{n,j}(\boldsymbol{\beta}_j) = (\mathbb{P}_n - P)\left\{\int_0^\tau E(t, \boldsymbol{\beta}_j)\left[d\tilde{N}_j(t; \hat{\boldsymbol{\gamma}}_n) - d\tilde{N}_j(t; \boldsymbol{\gamma}_0)\right]\right\}.$$

The terms $\mathbf{A}_{n,j}$ coverge to $\mathbf{0}$ almost surely by the almost sure consistency of $\hat{\boldsymbol{\gamma}}_n$ and conditions C3 and C5. The same is true for the term $\mathbf{B}_{n,j}$ as a consequence of the strong law of large numbers and the boundedness condition C5. Next, it is easy to argue that the classes of functions $\{Y(t)\exp(\boldsymbol{\beta}^T\mathbf{Z}) : t \in [0, \tau], \boldsymbol{\beta} \in \mathcal{B}_j\}$ and $\{\mathbf{Z}Y(t)\exp(\boldsymbol{\beta}^T\mathbf{Z}) : t \in [0, \tau], \boldsymbol{\beta} \in \mathcal{B}_j\}$ are Donsker and thus also

Glivenko-Cantelli, which combined with condition C1 and strong consistency of $\hat{\gamma}_n$ lead to the fact that $\sup_{\boldsymbol{\beta}_j \in \mathcal{B}_j} \|C_{n,j}(\boldsymbol{\beta}_j)\| \overset{as*}{\to} \mathbf{0}$. Finally, for $\mathbf{D}_{n,j}(\boldsymbol{\beta}_j)$ consider the class of functions

$$\mathcal{L}_j^{(l)} = \left\{ [R\Delta_j + (1-R)\pi_j(\mathbf{W}, \boldsymbol{\gamma}_0)] \int_0^\tau E^{(l)}(t, \boldsymbol{\beta}) dN(t) : \boldsymbol{\beta} \in \mathcal{B}_j \right\},$$

where

$$E^{(l)}(t, \boldsymbol{\beta}_j) = \frac{P[Z^{(l)} Y(t) \exp(\boldsymbol{\beta}_j^T \mathbf{Z})]}{P[Y(t) \exp(\boldsymbol{\beta}_j^T \mathbf{Z})]},$$

with $Z^{(l)}$ being the lth component of $\mathbf{Z}$. Based on the Lipschitz continuity property of $E^{(l)}(t, \boldsymbol{\beta}_j)$ we can argue that for any finitely discrete probability measure Q and any $\boldsymbol{\beta}_1, \boldsymbol{\beta}_2 \in \mathcal{B}_j$ and $f_{\boldsymbol{\beta}_1}^{(l)}, f_{\boldsymbol{\beta}_2}^{(l)} \in \mathcal{L}_j$,

$$\begin{aligned} \left\| f_{\boldsymbol{\beta}_1}^{(l)} - f_{\boldsymbol{\beta}_2}^{(l)} \right\|_{Q,2} &\le \left\| [R\Delta_j + (1-R)\pi_j(\mathbf{W}, \boldsymbol{\gamma}_0)] \int_0^\tau \left| E^{(l)}(t, \boldsymbol{\beta}_1) - E^{(l)}(t, \boldsymbol{\beta}_2) \right| dN(t) \right\|_{Q,2} \\ &\le \left\| \int_0^\tau \left| E^{(l)}(t, \boldsymbol{\beta}_1) - E^{(l)}(t, \boldsymbol{\beta}_2) \right| dN(t) \right\|_{Q,2} \\ &\le K \|\boldsymbol{\beta}_1 - \boldsymbol{\beta}_2\| \, \|N(\tau)\|_{Q,2} \end{aligned}$$

Hence for any $\boldsymbol{\beta} \in \mathcal{B}_j$ there exists a $\boldsymbol{\beta}_i$, $i = 1, \ldots, N(\epsilon, \mathcal{B}_j, \|\cdot\|)$, such that $\|\boldsymbol{\beta}_i - \boldsymbol{\beta}\| < \epsilon$. Consequently, for any $f_{\boldsymbol{\beta}}^{(l)} \in \mathcal{L}_{1j}^{(l)}$ there exists an $f_{\boldsymbol{\beta}_i}$ such that

$$\left\| f_{\boldsymbol{\beta}_i}^{(l)} - f_{\boldsymbol{\beta}}^{(l)} \right\|_{Q,2} \le K\epsilon \equiv \epsilon',$$

and thus we can cover the whole $\mathcal{L}_j^{(l)}$ with $N(\epsilon, \mathcal{B}_j, \|\cdot\|)$ $L_2(Q)$ ϵ'-balls centered at $f_{\boldsymbol{\beta}_i}$. By the minimality of the covering number we have that

$$N(\epsilon', \mathcal{L}_j^{(l)}, L_2(Q)) \le N(\epsilon, \mathcal{B}_j, \|\cdot\|),$$

which implies that $\mathcal{L}_j^{(l)}$ satisfies the uniform entropy bound given by 2.5.1 in [4]. Additionally, $\mathcal{L}_j^{(l)}$ can be shown to be a pointwise measurable class using similar arguments to those presented in page 142 in [2]. Therefore, $\mathcal{L}_j^{(l)}$ is Donsker by proposition 8.11 of [2] and Theorem 2.5.2 of [4]. This fact along with the strong consistency of $\hat{\gamma}_n$ and the conditions C1, C3 and C5 lead to $\sup_{\boldsymbol{\beta}_j \in \mathcal{B}_j} \|\mathbf{D}_n(\boldsymbol{\beta}_j)\| \overset{as*}{\to} 0$, and thus

$$\sup_{\boldsymbol{\beta}_j \in \mathcal{B}_j} \|\mathbf{G}_{n,j}(\boldsymbol{\beta}_j; \hat{\boldsymbol{\gamma}}_n) - \mathbf{G}_j(\boldsymbol{\beta})\| \overset{as*}{\to} 0.$$

This fact along with condition C6, which ensures that $\mathbf{G}_j(\boldsymbol{\beta})$ is concave and thus it has a unique root, leads to the strong consistency of $\hat{\boldsymbol{\beta}}_{n,j}$ for all $j = 1, \ldots, k$, by Theorem 2.10 of [2].

Next, for the proof of consistency of $\hat{\Lambda}_{n,j}(t)$, we have the following expansion

$$\hat{\Lambda}_{n,j}(t) - \Lambda_{0,j}(t) = A^{\star}_{n,j}(t) + B^{\star}_{n,j}(t), \qquad (1)$$

were.

$$A^{\star}_{n,j}(t) = \mathbb{P}_n \left\{ (1-R)[\pi_j(\mathbf{W}, \hat{\boldsymbol{\gamma}}_n) - \pi_j(\mathbf{W}, \boldsymbol{\gamma}_0)] \int_0^t I[\mathbb{P}_n Y(s) > 0] \frac{dN(s)}{\mathbb{P}_n[Y(s) e^{\hat{\beta}^T_{n,j}\mathbf{Z}}]} \right\}$$

and

$$B^{\star}_{n,j}(t) = \left\{ \int_0^t \frac{\mathbb{P}_n[d\tilde{N}_j(s;\boldsymbol{\gamma}_0)]}{\mathbb{P}_n[Y(s)e^{\hat{\boldsymbol{\beta}}^T_{n,j}\mathbf{Z}}]} - \int_0^t \frac{P[d\tilde{N}_j(s;\boldsymbol{\gamma}_0)]}{P[Y(s)e^{\boldsymbol{\beta}^T_{0,j}\mathbf{Z}}]} \right\}$$

for all $j = 1, \ldots, k$. Using the almost sure consitency of $\hat{\boldsymbol{\gamma}}_n$ and conditions C3 and C5 it can be easily argued that $\|A^{\star}_{n,j}(t)\|_\infty \overset{as}{\to} 0$, by the continuous mapping theorem. The uniform outer almost sure convergence of $B^{\star}_{n,j}(t)$ to 0 follows by an expansion and arguments similar to those provided in page 57 of [2]. This completes the proof of the uniform outer almost sure consistency of $\hat{\Lambda}_{n,j}(t)$ for all $j = 1, \ldots, k$.

2.2 Proof of Theorem 2

Since the estimator $\hat{\boldsymbol{\beta}}_{n,j}$ satisfies $\mathbf{G}_{n,j}(\hat{\boldsymbol{\beta}}_{n,j}; \hat{\boldsymbol{\gamma}}_n) = \mathbf{0}$, it follows that

$$\begin{aligned} \mathbf{0} &= \sqrt{n}\mathbf{G}_{n,j}(\hat{\boldsymbol{\beta}}_{n,j}; \hat{\boldsymbol{\gamma}}_n) \\ &= \sqrt{n}\left[\mathbf{G}_{n,j}(\hat{\boldsymbol{\beta}}_{n,j}; \hat{\boldsymbol{\gamma}}_n) - \mathbf{G}_{n,j}(\hat{\boldsymbol{\beta}}_{n,j}; \boldsymbol{\gamma}_0)\right] \\ &\quad + \sqrt{n}\mathbf{G}_{n,j}(\hat{\boldsymbol{\beta}}_{n,j}; \boldsymbol{\gamma}_0). \end{aligned} \qquad (2)$$

The first term of (2) can be expressed, after some algebra, as

$$\sqrt{n}\left[\mathbf{G}_{n,j}(\hat{\boldsymbol{\beta}}_{n,j}; \hat{\boldsymbol{\gamma}}_n) - \mathbf{G}_{n,j}(\hat{\boldsymbol{\beta}}_{n,j}; \boldsymbol{\gamma}_0)\right] = \mathbf{A}'_{n,j} + \mathbf{B}'_{n,j} + \mathbf{C}'_{n,j} + \mathbf{D}'_{n,j}.$$

where

$$\mathbf{A}'_{n,j} = \sqrt{n}(\mathbb{P}_n - P)\left\{\mathbf{Z}N(\tau)(1-R)\left[\pi_j(\mathbf{W}, \hat{\boldsymbol{\gamma}}_n) - \pi_j(\mathbf{W}, \boldsymbol{\gamma}_0)\right]\right\},$$

$$\mathbf{B}'_{n,j} = \sqrt{n}(\mathbb{P}_n - P)\left\{[\pi_j(\mathbf{W}, \hat{\boldsymbol{\gamma}}_n) - \pi_j(\mathbf{W}; \boldsymbol{\gamma}_0)] \int_0^\tau E(t, \boldsymbol{\beta}_{0,j}) dN(t)\right\},$$

$$\mathbf{C}'_{n,j} = \sqrt{n}\mathbb{P}_n\left\{\int_0^\tau \left[E_n(t, \hat{\boldsymbol{\beta}}_{n,j}) - E(t, \boldsymbol{\beta}_{0,j})\right] d\left[\tilde{N}_j(t; \hat{\boldsymbol{\gamma}}_n) - \tilde{N}_j(t; \boldsymbol{\gamma}_0)\right]\right\}$$

and

$$\mathbf{D}'_{n,j} = \left(P\left\{(1-R)\int_0^\tau [\mathbf{Z} - E(t, \boldsymbol{\beta}_{0,j})] dN(t) \dot{\pi}_j(\mathbf{W}, \boldsymbol{\gamma}_0)^T\right\}\right) \times \sqrt{n}(\hat{\boldsymbol{\gamma}}_n - \boldsymbol{\gamma}_0).$$

It is straighforward to argue that the class $\{\pi_j(\mathbf{W},\boldsymbol{\gamma}) : \boldsymbol{\gamma} \in \mathit{\Gamma}\}$ is Donsker due to the Lipschitz continuity in $\boldsymbol{\gamma}$, as a result of condition C3, which implies that the class

$$\left\{\mathbf{Z}N(\tau)[\pi_j(\mathbf{W},\boldsymbol{\gamma}) - \pi_j(\mathbf{W},\boldsymbol{\gamma}_0)] : \boldsymbol{\gamma} \in \mathit{\Gamma}\right\}$$

is also Donsker. Since $P\left\{\mathbf{Z}N(\tau)\left[\pi_j(\mathbf{W},\boldsymbol{\gamma}) - \pi_j(\mathbf{W},\boldsymbol{\gamma}_0)\right]\right\}^2 \to \mathbf{0}$ as $\boldsymbol{\gamma} \to \boldsymbol{\gamma}_0$, and by the consistency of $\hat{\boldsymbol{\gamma}}_n$, it follows by Corollary 2.3.12 of [4] that $\mathbf{A}'_{n,j} \overset{p}{\to} \mathbf{0}$. Similar arguments along with the Donsker property for the class

$$\left\{[\pi_j(\mathbf{W},\boldsymbol{\gamma}) - \pi_j(\mathbf{W},\boldsymbol{\gamma}_0)]\int_0^\tau E(t,\boldsymbol{\beta}_{0,j})dN(t) : \boldsymbol{\gamma} \in \mathit{\Gamma}\right\}$$

can be used to show that $\mathbf{B}'_{n,j} \overset{p}{\to} \mathbf{0}$. Next, using similar arguments to that used in the proof of consistency of $\hat{\boldsymbol{\beta}}_{n,j}$, it can be shown that $\|E_n(t,\hat{\boldsymbol{\beta}}_{n,j}) - E(t,\boldsymbol{\beta}_{0,j})\|_\infty \overset{as*}{\to} \mathbf{0}$. The boundedness conditions imply the uniform boundedness of the variation of the process (of time) $E_n(t,\hat{\boldsymbol{\beta}}_{n,j}) - E(t,\boldsymbol{\beta}_{0,j})$. Additionally, it can be easily argued that $\sqrt{n}\mathbb{P}_n[\tilde{N}_j(t;\hat{\boldsymbol{\gamma}}_n) - \tilde{N}_j(t;\boldsymbol{\gamma}_0)]$ converges weakly to a tight zero-mean Gaussian process, and thus it follows that $\mathbf{C}'_{n,j} \overset{p}{\to} \mathbf{0}$ by Lemma 4.2 of [2]. Therefore, the first term in (2) is equal to $\mathbf{D}'_{n,j} + o_p(1)$. Next, a Taylor expansion of the second term of (2) around $\boldsymbol{\beta}_{0,j}$ and some algebra lead to

$$\begin{aligned}\sqrt{n}\mathbf{G}_{n,j}(\hat{\boldsymbol{\beta}}_{n,j};\boldsymbol{\gamma}_0) &= \sqrt{n}\mathbf{G}_{n,j}(\boldsymbol{\beta}_{0,j};\boldsymbol{\gamma}_0) - \mathbf{H}_j(\boldsymbol{\beta}_{0,j})\sqrt{n}(\hat{\boldsymbol{\beta}}_{n,j} - \boldsymbol{\beta}_{0,j}) \\ &\quad + o_p(1 + \sqrt{n}\|\hat{\boldsymbol{\beta}}_{n,j} - \boldsymbol{\beta}_{0,j}\|). \end{aligned} \tag{3}$$

By condition C6, $\mathbf{H}_j(\boldsymbol{\beta}_j)$ is invertible and thus there exists a constant $K > 0$ such that for any $\boldsymbol{\beta}_j \in \mathcal{B}$ we have

$$\|\mathbf{H}_j(\boldsymbol{\beta}_{0,j})(\boldsymbol{\beta}_j - \boldsymbol{\beta}_{0,j})\| \geq K\|\boldsymbol{\beta}_j - \boldsymbol{\beta}_{0,j}\|, \quad j = 1,\ldots,k.$$

Now, applying a Taylor expansion of $\mathbf{G}_j(\boldsymbol{\beta}_j;\boldsymbol{\gamma}_0)$ around $\boldsymbol{\beta}_{0,j}$ leads to

$$\|\mathbf{G}_{n,j}(\boldsymbol{\beta}_j;\boldsymbol{\gamma}_0) - \mathbf{G}_j(\boldsymbol{\beta}_{0,j};\boldsymbol{\gamma}_0)\| \geq K\|\boldsymbol{\beta}_j - \boldsymbol{\beta}_{0,j}\| + o(\|\boldsymbol{\beta}_j - \boldsymbol{\beta}_{0,j}\|), \tag{4}$$

for $j = 1,\ldots,k$. Next, it can be easily shown that

$$\begin{aligned}\sqrt{n}[\mathbf{G}_{n,j}(\hat{\boldsymbol{\beta}}_{n,j};\boldsymbol{\gamma}_0) - \mathbf{G}_{n,j}(\boldsymbol{\beta}_{0,j};\boldsymbol{\gamma}_0)] &= -\sqrt{n}(\mathbb{P}_n - P)\int_0^\tau [\mathbf{Z} - E(t,\boldsymbol{\beta}_{0,j})]d\tilde{N}_j(t;\boldsymbol{\gamma}_0) \\ &\quad + o_p(1 + \sqrt{n}\|\hat{\boldsymbol{\beta}}_{n,j} - \boldsymbol{\beta}_{0,j}\|) + o_p(1) \\ &= O_p(1) + o_p(1 + \sqrt{n}\|\hat{\boldsymbol{\beta}}_{n,j} - \boldsymbol{\beta}_{0,j}\|) + o_p(1).\end{aligned}$$

Combining the above equation with (4) leads to

$$\sqrt{n}\|\hat{\boldsymbol{\beta}}_{n,j} - \boldsymbol{\beta}_{0,j}\|[K + o_p(1)] \leq O_p(1) + o_p(1 + \sqrt{n}\|\hat{\boldsymbol{\beta}}_{n,j} - \boldsymbol{\beta}_{0,j}\|)$$

and thus $\sqrt{n}\|\hat{\boldsymbol{\beta}}_{n,j} - \boldsymbol{\beta}_{0,j}\| = O_p(1)$. Therefore, the remainder term in (3) is $o_p(1)$. Next, for the second term, it is straightforward to show that $P\tilde{M}_j(t;\boldsymbol{\beta}_{0,j},\boldsymbol{\gamma}_0) = 0$ where

$$\tilde{M}_{ij}(t;\boldsymbol{\beta}_{0,j},\boldsymbol{\gamma}_0) = \tilde{N}_{ij}(t;\boldsymbol{\gamma}_0) - \int_0^t Y_i(s)\exp(\boldsymbol{\beta}_{0,j}^T\mathbf{Z}_i)d\Lambda_{0,j}(s),$$

for all $t \in [0,\tau]$ and $j = 1,\dots,k$. Also, the class $\{\tilde{M}_j(t;\boldsymbol{\beta}_{0,j},\boldsymbol{\gamma}_0) : t \in [0,\tau]\}$, for all $j = 1,\dots,k$, is Donsker since both $\tilde{N}_j(t;\boldsymbol{\gamma}_0)$ and $\int_0^t Y(s)\exp(\boldsymbol{\beta}_{0,j}^T\mathbf{Z})d\Lambda_{0,j}(s)$ form Donsker classes indexed by $t \in [0,\tau]$. The Donsker property for the latter class follows from the fact that it is formed by bounded, by conditions C1, C2 and C5, monotone cadlag processes and Lemma 4.1 in [2]. Therefore,

$$\sqrt{n}G_{n,j}(\beta_{0,j};\gamma_0) = \sqrt{n}\mathbb{P}_n\int_0^\tau \{Z - E(t,\beta_{0,j})\}d\tilde{M}_j(t;\beta_{0,j},\gamma_0) + o_p(1),$$

by Lemma 4.2 of [2]. Taking all the pieces together along with conditions C4 and C6 we obtain

$$\sqrt{n}(\hat{\boldsymbol{\beta}}_{n,j} - \boldsymbol{\beta}_{0,j}) = \sqrt{n}\mathbb{P}_n(\boldsymbol{\psi}_j + \mathbf{R}_j\boldsymbol{\omega}) + o_p(1), \quad j = 1,\dots,k,$$

where the influence functions were defined in the main text before stating Theorem 2. Now, to show the consistency in probability of the covariance esimator $\hat{\boldsymbol{\Sigma}}_j = \mathbb{P}_n(\hat{\boldsymbol{\psi}}_j + \hat{\mathbf{R}}_j\hat{\boldsymbol{\omega}})^{\otimes 2}$ note that

$$\hat{\boldsymbol{\Sigma}}_j = \mathbf{H}_{n,j}^{-1}(\hat{\boldsymbol{\beta}}_{n,j})\left[\mathbb{P}_n(\hat{\boldsymbol{\psi}}_j' + \hat{\mathbf{R}}_j'\hat{\omega})^{\otimes 2}\right]\mathbf{H}_{n,j}^{-1}(\hat{\boldsymbol{\beta}}_{n,j}),$$

where $\hat{\boldsymbol{\psi}}_j' = \mathbf{H}_{n,j}(\hat{\boldsymbol{\beta}}_{n,j})\hat{\boldsymbol{\psi}}_j$ and $\hat{\mathbf{R}}_j' = \mathbf{H}_{n,j}(\hat{\boldsymbol{\beta}}_{n,j})\hat{\mathbf{R}}_j$. Similarly,

$$\boldsymbol{\Sigma}_j = \mathbf{H}_j^{-1}(\boldsymbol{\beta}_{0,j})\left[P(\boldsymbol{\psi}_j' + \mathbf{R}_j'\omega)^{\otimes 2}\right]\mathbf{H}_{n,j}^{-1}(\boldsymbol{\beta}_{0,j}),$$

where $\boldsymbol{\psi}_j' = \mathbf{H}_j(\boldsymbol{\beta}_{0,j})\boldsymbol{\psi}_j$ and $\mathbf{R}_j' = \mathbf{H}_j(\boldsymbol{\beta}_{0,j})\mathbf{R}_j$. By Theorem 1, conditions C1-C6, and standard arguments for the Cox model can be used to show that $\mathbf{H}_{n,j}^{-1}(\hat{\boldsymbol{\beta}}_{n,j}) \xrightarrow{p} \mathbf{H}_j^{-1}(\boldsymbol{\beta}_{0,j})$. Now, the fact that $\sup_{t\in[0,\tau]}\|E_n(t,\hat{\boldsymbol{\beta}}_{n,j}) - E(t,\boldsymbol{\beta}_{0,j})\| \xrightarrow{as*} 0$ as **it** was argued earlier, conditions C3 and C4, and the weak law of large numbers lead to the conclusion that $\hat{\mathbf{R}}_j' \xrightarrow{p} \mathbf{R}_j'$. Finally, conditions C3-C5, Theorem 1, Lemma 4.2 in [3], and some algebra can be used to show that $\mathbb{P}_n(\hat{\boldsymbol{\psi}}_j' + \hat{\mathbf{R}}_j'\hat{\omega})^{\otimes 2} \xrightarrow{p} P(\boldsymbol{\psi}_j' + \mathbf{R}_j'\omega)^{\otimes 2}$, and therefore $\hat{\boldsymbol{\Sigma}}_j \xrightarrow{p} \boldsymbol{\Sigma}_j$.

2.3 Proof of Theorem 3

By Taylor expansion and the consistency of $\hat{\boldsymbol{\beta}}_{n,j}$ and $\hat{\boldsymbol{\gamma}}_n$, the first term in the right side of expansion (1) can be shown to be

$$A_{n,j}^\star(t) = \left(P\left\{(1-R)\dot{\pi}_j(\mathbf{W},\boldsymbol{\gamma}_0)\int_0^t \frac{dN(s)}{P[Y(s)e^{\boldsymbol{\beta}_{0,j}^T\mathbf{Z}}]}\right\}\right)^T(\hat{\boldsymbol{\gamma}}_n - \boldsymbol{\gamma}_0) + o_p(n^{-1/2}).$$

Using similar analysis to that presented in page 57 of [2] and the regularity condition C4 regarding $\hat{\gamma}_n$ leads to the conclusion that the second term in (1) is

$$B^\star_{n,j}(t) = \mathbb{P}_n \int_0^t \frac{d\tilde{M}_j(s;\boldsymbol{\beta}_{0,j},\boldsymbol{\gamma}_0)}{P[Y(s)e^{\boldsymbol{\beta}_{0,j}^T\mathbf{Z}}]} - (\hat{\boldsymbol{\beta}}_{n,j} - \boldsymbol{\beta}_{0,j})^T \int_0^t E(s,\boldsymbol{\beta}_{0,j})d\Lambda_{0,j}(s) + o_p(n^{-1/2}).$$

Therefore

$$\sqrt{n}\left[\hat{\Lambda}_{n,j}(t) - \Lambda_{0,j}(t)\right] = \sqrt{n}\mathbb{P}_n[\phi_j(t) + \mathbf{R}^\star_j(t)\boldsymbol{\omega}] + o_p(1).$$

Condition C4, the fact that $\mathbf{R}^\star_j(t)$ is non-random and standard arguments related to $\phi_j(t)$ [2] imply that the class of influence functions is Donsker, and thus the right-hand side of the above equality converges weakly to a tight mean-zero Gaussian process with covariance function $P\{[\phi_j(t) + \mathbf{R}^\star_j(t)\boldsymbol{\omega}][\phi_j(s) + \mathbf{R}^\star_j(s)\boldsymbol{\omega}]\}$. It can be shown using standard results for the Cox model [2], the facts that $\hat{\boldsymbol{\Sigma}}_j \xrightarrow{p} \boldsymbol{\Sigma}_j$ and $\sup_{t\in[0,\tau]} \|\hat{\mathbf{R}}^\star_j(t) - \mathbf{R}^\star_j(t)\| = o_p(1)$ (the latter will be proved in the next paragraph), and condition C4 that

$$\sup_{t\in[0,\tau]} \mathbb{P}_n \left\{[\hat{\phi}_{ij}(t) - \hat{\mathbf{R}}^\star_j(t)\hat{\boldsymbol{\omega}}] - [\phi_j(t) + \mathbf{R}^\star_j(t)\boldsymbol{\omega}]\right\}^2 = o_p(1).$$

The Donsker property of the class of influence functions $\{\phi_j(t) + \mathbf{R}^\star_j(t)\boldsymbol{\omega} : t \in [0,\tau]\}$ and the square integrability of the influence functions as a result of the boundedness conditions lead to the conclusion that $\mathbb{P}_n\{[\hat{\phi}_j(t) + \hat{\mathbf{R}}^\star_j(t)\hat{\boldsymbol{\omega}}][\hat{\phi}_j(s) + \hat{\mathbf{R}}^\star_j(s)\hat{\boldsymbol{\omega}}]\}$, $t, s \in [0,\tau]$, is a uniformly consistent (in probability) estimator of the covariance function $P\{[\phi_j(t) + \mathbf{R}^\star_j(t)\boldsymbol{\omega}][\phi_j(s) + \mathbf{R}^\star_j(s)\boldsymbol{\omega}]\}$ by Lemma 9.28 in [2].

Now, in order to show the final statement of Theorem 3 let $\hat{W}_{n,j}(t) = \sqrt{n}\mathbb{P}_n[\hat{\phi}_j(t) + \hat{\mathbf{R}}^\star_j(t)\hat{\boldsymbol{\omega}}]\xi$ and $\tilde{W}_{n,j}(t) = \sqrt{n}\mathbb{P}_n[\phi_j(t) + \mathbf{R}^\star_j(t)\boldsymbol{\omega}]\xi$. It follows from the Donsker property of the class of influence functions $\{\phi_j(t) + \mathbf{R}^\star_j(t)\boldsymbol{\omega} : t \in [0,\tau]\}$ and the conditional multiplier central limit theorem [4] that $\tilde{W}_{n,j}(t)$ converges weakly conditional on the data to the same limiting process as that of $\sqrt{n}[\hat{\Lambda}_{n,j}(t) - \Lambda_{0,j}(t)]$ (unconditionally). In order to complete the proof of the final statement we need to show that

$$\|\hat{W}_{n,j}(t) - \tilde{W}_{n,j}(t)\|_\infty = o_p(1) \qquad j = 1,\ldots,k,$$

since this implies that $\hat{W}_{n,j}(t)$ and $\tilde{W}_{n,j}(t)$ converge weakly (unconditionally) to the same limiting process. It can be easily seen that

$$\|\hat{W}_{n,j}(t) - \tilde{W}_{n,j}(t)\|_\infty \leq A''_{n,j} + B''_{n,j} + C''_{n,j}$$

where

$$A''_{n,j} = \|\sqrt{n}\mathbb{P}_n[\hat{\phi}_j(t) - \phi_j(t)]\xi\|_\infty,$$

$$B''_{n,j} = \sup_{t\in[0,\tau]} \left\|\hat{\mathbf{R}}^\star_j(t) - \mathbf{R}^\star_j(t)\right\| \times \left(\left\|\sqrt{n}\mathbb{P}_n[\hat{\boldsymbol{\omega}} - \boldsymbol{\omega}]\xi\right\| + \left\|\sqrt{n}\mathbb{P}_n\boldsymbol{\omega}\xi\right\|\right),$$

$$C''_{n,j} = \sup_{t\in[0,\tau]} \left\|\mathbf{R}^{\star}_j(t)\right\| \times \left\|\sqrt{n}\mathbb{P}_n[\hat{\boldsymbol{\omega}} - \boldsymbol{\omega}]\xi\right\|.$$

Using the same arguments as those used in the proof of Theorem 4 in [3] along with conditions C3 and C4 leads to the conclusion that $A''_{n,j} = o_p(1)$. Next, considering the term $B''_{n,j}$, we have that $\|\sqrt{n}\mathbb{P}_n[\hat{\boldsymbol{\omega}} - \boldsymbol{\omega}]\xi\| = o_p(1)$ by arguments similar to those used in the proof of Lemma A.3 in [3]. Additionally, $\|\sqrt{n}\mathbb{P}_n\boldsymbol{\omega}\xi\| = O_p(1)$ by C4 and the central limit theorem. The first factor of $B''_{n,j}$ is

$$\begin{aligned}\sup_{t\in[0,\tau]} \left\|\hat{\mathbf{R}}^{\star}_j(t) - \mathbf{R}^{\star}_j(t)\right\| \leq & \sup_{t\in[0,\tau]} \left\|\mathbb{P}_n\left[\dot{\pi}_j(\mathbf{W},\hat{\boldsymbol{\gamma}}_n) - \dot{\pi}_j(\mathbf{W},\boldsymbol{\gamma}_0)\right]\int_0^t \frac{dN(s)}{\mathbb{P}_n Y(s) e^{\hat{\boldsymbol{\beta}}^T_{n,j}\mathbf{Z}}}\right\| \\ & + \sup_{t\in[0,\tau]} \left\|\mathbb{P}_n\dot{\pi}_j(\mathbf{W},\boldsymbol{\gamma}_0)\int_0^t \left[\frac{1}{\mathbb{P}_n Y(s) e^{\hat{\boldsymbol{\beta}}^T_{n,j}\mathbf{Z}}} - \frac{1}{PY(s)e^{\boldsymbol{\beta}^T_{0,j}\mathbf{Z}}}\right] dN(s)\right\| \\ & + \sup_{t\in[0,\tau]} \left\|(\mathbb{P}_n - P)\dot{\pi}_j(\mathbf{W},\boldsymbol{\gamma}_0)\int_0^t \frac{1}{PY(s)e^{\boldsymbol{\beta}^T_{0,j}\mathbf{Z}}} dN(s)\right\|. \qquad (5)\end{aligned}$$

By conditions C3, C4, C5 and the continuous mapping theorem

$$\max_i \|\dot{\pi}_j(\mathbf{W}_i,\hat{\boldsymbol{\gamma}}_n) - \dot{\pi}_j(\mathbf{W}_i,\boldsymbol{\gamma}_0)\| = o_{as}(1).$$

Also, by Theorem 1 and conditions C1, C2, C5

$$\left\|\frac{1}{\mathbb{P}_n Y(t) e^{\hat{\boldsymbol{\beta}}^T_{n,j}\mathbf{Z}}}\right\|_\infty = \left\|\frac{1}{PY(t)e^{\boldsymbol{\beta}^T_{0,j}\mathbf{Z}} + o_{as*}(1)}\right\|_\infty = O_{as*}(1),$$

and therefore the first term in the right side of (5) is $o_{as*}(1)$. By conditions C3 and C5, which lead to the conclusion that

$$\max_i \|\dot{\pi}_j(\mathbf{W}_i,\boldsymbol{\gamma}_0)\| = O_p(1),$$

and the fact that

$$\left\|\frac{1}{\mathbb{P}_n Y(t) e^{\hat{\boldsymbol{\beta}}^T_{n,j}\mathbf{Z}}} - \frac{1}{PY(t)e^{\boldsymbol{\beta}^T_{0,j}\mathbf{Z}}}\right\|_\infty = o_{as^\star}(1),$$

as a result of Theorem 1, the Donsker property of the class $\{Y(t) : t \in [0,\tau]\}$, conditions C2 and C5, and some algebra, it follows that the second term in the right side of (5) is also $o_p(1)$. Finally, consider the classes of functions $\mathcal{F} = \{N(t) : t \in [0,\tau]\}$ and

$$\mathcal{L}_{j,1} = \left\{ f_{t,j} = \dot{\pi}_j(\mathbf{W},\boldsymbol{\gamma}_0)\int_0^t \frac{dN(s)}{P\left[Y(s)\exp(\boldsymbol{\beta}^T_{0,j}\mathbf{Z})\right]} : t \in [0,\tau] \right\}.$$

For any finitely discrete probability measure Q and any $t_1, t_2 \in [0,\tau]$ we have

$$\|f_{t_1,j} - f_{t_2,j}\|_{Q,2} \leq \left\| \dot{\pi}_j(\mathbf{W}, \boldsymbol{\gamma}_0) \int_{t_1}^{t_2} \frac{dN(s)}{P\left[Y(s)\exp(\boldsymbol{\beta}_{0,j}^T \mathbf{Z})\right]} \right\|_{Q,2}$$
$$\leq K\|N(t_2) - N(t_1)\|_{Q,2},$$

by the boundedness of $\dot{\pi}_j(\mathbf{W}, \boldsymbol{\gamma}_0)$. Consequently, for any $\epsilon > 0$ and $f_{t,j} \in \mathcal{L}_{j,1}$ with $t \in [0,\tau]$ there exists a $f_{t_i,j}$, $i = 1, \ldots, N(\epsilon, \mathcal{F}, L_2(Q))$, such that $\|f_{t,j} - f_{t_i,j}\|_{Q,2} \leq K\epsilon \equiv \epsilon'$ and thus the class $\mathcal{L}_{j,1}$ can be covered by $N(\epsilon, \mathcal{F}, L_2(Q))$ $L_2(Q)$ ϵ'-balls centered at $f_{t_i,j}$. Since $\mathcal{F}$ is Donsker, $\mathcal{L}_{j,1}$ satisfies the uniform entropy bound given by 2.5.1 in [4]. It can be easily argued that the class $\mathcal{L}_{j,1}$ is pointwise measurable [4,2]. Now, by proposition 8.11 of [2] and Theorem 2.5.2 of [4], it follows that $\mathcal{L}_{j,1}$ is Donsker. This implies the Glivenko-Cantelli property of that class and thus it follows that the last term in the right side of (5) is $o_p(1)$. Therefore, $\sup_{t\in[0,\tau]} \|\hat{\mathbf{R}}_j^{\star}(t) - \mathbf{R}_j^{\star}(t)\| = o_p(1)$ and thus $B''_{n,j} = o_p(1)$. Finally, $C''_{n,j} = o_p(1)$ by similar arguments to those used for the term $B''_{n,j}$, and thus $\|\hat{W}_{n,j}(t) - \tilde{W}_{n,j}(t)\|_\infty = o_p(1)$ for all $j = 1, \ldots, k$, which completes the proof of the final statement of Theorem 3.

2.4 Proof of Theorem 4

The asymptotic expression in Theorem 4 follows from a decomposition of $\sqrt{n}[\hat{F}_{n,j}(t;\mathbf{z}_0) - F_{0,j}(t;\mathbf{z}_0)]$ similar to that used in [1], our Theorems 1, 2 and 3, Lemma 4.2 in [2] and integration by parts. To show the Donsker property of the class $\Phi_j(\mathbf{z}_0) = \{\phi_j^F(t;\mathbf{z}_0) : t \in [0,\tau]\}$, for all $j = 1, \ldots, k$ and $\mathbf{z}_0$ in the bounded finite-dimensional space, we will use the following Lemma.

Lemma 1 *Let $g(t)$ be a fixed uniformly bounded function and $f(t) = f_1(t) - f_2(t)$ on $[0,\tau]$, with $\mathcal{F}_l = \{f_l(t) : t \in [0,\tau]\}$, $l = 1, 2$, being Donsker classes of measurable, non-decreasing and right-continuous functions. Then, the class*

$$\mathcal{F}_3 = \left\{ \int_0^t g(s)df(s) : t \in [0,\tau] \right\},$$

is Donsker.

Proof For any $t_1, t_2 \in [0,\tau]$ and any finitely discrete probability measure Q it follows that

$$\left\| \int_0^{t_1} g(s)df_l(s) - \int_0^{t_2} g(s)df_l(s) \right\|_{Q,2} \leq K\|f_l(t_1) - f_l(t_2)\|_{Q,2}, \qquad l = 1, 2,$$

where $K = \sup_{t\in[0,\tau]} |g(t)|$. Now, for any $t \in [0,\tau]$ there exists a $t_i \in [0,\tau]$, with $i = 1, \ldots, N(\epsilon, \mathcal{F}_l, L_2(Q))$ and $l = 1, 2$, such that $\|f_l(t_i) - f_l(t)\|_{Q,2} < \epsilon$. Therefore, for any $t \in [0,\tau]$ there exists a $t_i \in [0,\tau]$ such that $\|\phi_l(t_i) -$

$\phi_l(t)\|_{Q,2} < K\epsilon$, with $\phi_l \in \mathcal{F}_{3,l} = \left\{\int_0^t g(s)df_l(s) : f_l \in \mathcal{F}_l\right\}$, $l = 1,2$, and thus $\mathcal{F}_{3,l}$ can be covered by $N(\epsilon, \mathcal{F}_l, L_2(Q))$ $L_2(Q)$ $K\epsilon$-balls. Therefore, by the Donsker property of the classes $\mathcal{F}_l$, $l = 1,2$, the classes $\mathcal{F}_{3,l}$, $l = 1,2$, satisfy the uniform entropy bound. Additionally, it can be argued that $\mathcal{F}_{3,1}$ and $\mathcal{F}_{3,2}$ are both pointwise measurable. Consequently, by proposition 8.11 of [2] and Theorem 2.5.2 of [4], it follows that $\mathcal{F}_{3,1}$ and $\mathcal{F}_{3,2}$ are Donsker. Finally, the Donsker property of $\mathcal{F}_3$ is a consequence of Corollary 9.31 of [2], since $\mathcal{F}_3$ is formed by differences of functions that belong to Donsker classes.

The fact that $\phi^{\Lambda}_{ij}(t;\mathbf{z}_0)$ can be written as the difference of two non-decreasing right-continuous functions which both belong to a Donsker class, along with Lemma 1 above and integration by parts can be used to show the Donsker property of $\Phi_j(\mathbf{z}_0)$, for all $j = 1,\dots,k$ and $\mathbf{z}_0$ in the bounded covariate space. It can be shown using Theorems 1-3, the fact that $\sup_{t\in[0,\tau]} \mathbb{P}_n\{[\hat{\phi}_{ij}(t) - \hat{\mathbf{R}}^\star_j(t)\hat{\boldsymbol{\omega}}] - [\phi_j(t)+\mathbf{R}^\star_j(t)\boldsymbol{\omega}]\}^2 = o_p(1)$ as argued in the proof of Theorem 3, conditions C1-C5, and integration by parts, that $\sup_{t\in[0,\tau]} \mathbb{P}_n[\hat{\phi}^F_j(t;\mathbf{z}_0) - \phi^F_j(t;\mathbf{z}_0)]^2 = o_p(1)$. Futhermore, the Donsker property of $\Phi_j(\mathbf{z}_0)$, the square integrability of the influence functions $\phi^F_j(t;\mathbf{z}_0)$, and Lemma 9.28 in [2] lead to the conclusion that $\mathbb{P}_n\hat{\phi}^F_{ij}(t;\mathbf{z}_0)\hat{\phi}^F_{ij}(s;\mathbf{z}_0)$, $t,s \in [0,\tau]$, is a uniformly consistent (in probability) estimator of the covariance function $P\phi^F_j(t;\mathbf{z}_0)\phi^F_j(s;\mathbf{z}_0)$.

Now, in order to show the final statement of Theorem 4 let $\hat{W}_j(t;\mathbf{z}_0) = \sqrt{n}\mathbb{P}_n\hat{\phi}^F_j(t;\mathbf{z}_0)\xi$ and $\tilde{W}_j(t;\mathbf{z}_0) = \sqrt{n}\mathbb{P}_n\phi^F_j(t;\mathbf{z}_0)\xi$. It follows from the Donsker property of the class of influence functions $\Phi_j(\mathbf{z}_0)$ and the conditional multiplier central limit theorem [4] that $\tilde{W}^F_{n,j}(t;\mathbf{z}_0)$ converges weakly, conditionally on the data, to the same limiting process as that of $\sqrt{n}[\hat{F}_{n,j}(t;\mathbf{z}_0) - F_{0,j}(t;\mathbf{z}_0)]$ (unconditionally). Finally, we need to show that

$$\|\hat{W}^F_{n,j}(t;\mathbf{z}_0) - \tilde{W}^F_{n,j}(t;\mathbf{z}_0)\|_\infty = o_p(1) \quad j = 1,\dots,k.$$

Straightforward algebra and the fact that

$$\max_i \|\phi^{\Lambda}_{ij}(t;\mathbf{z}_0)\|_\infty = O_p(1), \quad j = 1,\dots,k$$

by the boundedness conditions, lead to the inequality

$$\|\hat{W}^F_{n,j}(t;\mathbf{z}_0) - \tilde{W}^F_{n,j}(t;\mathbf{z}_0)\|_\infty \le A'''_{n,j} + B'''_{n,j} + C'''_{n,j} + D'''_{n,j} + E'''_{n,j} + F'''_{n,j}$$

where

$$A'''_{n,j} = \left\|\int_0^t \exp\left[-\sum_{l=1}^k \hat{\Lambda}_{n,l}(s-;\mathbf{z}_0)\right] d\{\sqrt{n}\mathbb{P}_n[\hat{\phi}^{\Lambda}_j(s;\mathbf{z}_0) - \phi^{\Lambda}_j(s;\mathbf{z}_0)]\xi\}\right\|_\infty,$$

$$B'''_{n,j} = \left\|\int_0^t \left\{\exp\left[-\sum_{l=1}^k \hat{\Lambda}_{n,l}(s-;\mathbf{z}_0)\right] - \exp\left[-\sum_{l=1}^k \Lambda_{0,l}(s-;\mathbf{z}_0)\right]\right\} d[\sqrt{n}\mathbb{P}_n\phi^{\Lambda}_j(s;\mathbf{z}_0)\xi]\right\|_\infty,$$

$$C'''_{n,j} = \left\| \sum_{l=1}^{k} \int_0^t \left\{ \mathbb{P}_n \left[\hat{\phi}_l^{\Lambda}(s-;\mathbf{z}_0) - \phi_l^{\Lambda}(s-;\mathbf{z}_0) \right] \xi \right\} \exp\left[-\sum_{l=1}^{k} \hat{\Lambda}_{n,l}(s-;\mathbf{z}_0) \right] \right.$$
$$\left. \times d\{\sqrt{n}[\hat{\Lambda}_{n,j}(s;\mathbf{z}_0) - \Lambda_{0,j}(s;\mathbf{z}_0)]\} \right\|_{\infty},$$

$$D'''_{n,j} = \left\| \sum_{l=1}^{k} \int_0^t [\mathbb{P}_n \phi_l^{\Lambda}(s-;\mathbf{z}_0)\xi] \left\{ \exp\left[-\sum_{l=1}^{k} \hat{\Lambda}_{n,l}(s-;\mathbf{z}_0) \right] - \exp\left[-\sum_{l=1}^{k} \Lambda_{n,l}(s-;\mathbf{z}_0) \right] \right\} \right.$$
$$\left. \times d\{\sqrt{n}[\hat{\Lambda}_{n,j}(s;\mathbf{z}_0) - \Lambda_{0,j}(s;\mathbf{z}_0)]\} \right\|_{\infty},$$

$$E'''_{n,j} = \left\| \sum_{l=1}^{k} \int_0^t \left\{ \sqrt{n}\mathbb{P}_n \left[\hat{\phi}_l^{\Lambda}(s-;\mathbf{z}_0) - \phi_l^{\Lambda}(s-;\mathbf{z}_0) \right] \xi \right\} d\Lambda_{0,j}(s;\mathbf{z}_0) \right\|_{\infty},$$

$$F'''_{n,j} = O_p(1)|\sqrt{n}\mathbb{P}_n\xi| \left\| \int_0^t \left\{ \exp\left[-\sum_{l=1}^{k} \hat{\Lambda}_{n,l}(s-;\mathbf{z}_0) \right] - \exp\left[-\sum_{l=1}^{k} \Lambda_{0,l}(s-;\mathbf{z}_0) \right] \right\} d\Lambda_{0,j}(s;\mathbf{z}_0) \right\|_{\infty}.$$

Integration by parts, the fact that $\|\hat{W}_{n,j}(t) - \tilde{W}_{n,j}(t)\|_{\infty} = o_p(1)$ as it was shown in the proof of Theorem 3, Lemma A.3 in [3] and the boundedness conditions can be used to show that $A'''_{n,j} = o_p(1)$. Next, $B'''_{n,j} = o_p(1)$ by Theorem 1, the fact that $\sqrt{n}\mathbb{P}_n\phi_j^{\Lambda}(t;\mathbf{z}_0)\xi$ converges weakly to a tight mean zero Gaussian process due to the Donsker property of the class $\{\phi_j^{\Lambda}(t;\mathbf{z}_0) : t \in [0,\tau]\}$, and Lemma 4.2 in [2]. Using the fact that the integrand in $C'''_{n,j}$ converges uniformly to 0 in probability, Theorem 3, and Lemma 4.2 in [2] we can argue that $C'''_{n,j} = o_p(1)$. The same arguments can be used to show that $D'''_{n,j} = o_p(1)$. Additionally, $E'''_{n,j} = o_p(1)$ by the fact that the term inside the curly brackets is uniformly $o_p(1)$, as it was argued earlier, and condition C1. Finally, the fact that $\| \exp[-\sum_{l=1}^{k} \hat{\Lambda}_{n,l}(s-;\mathbf{z}_0)] - \exp[-\sum_{l=1}^{k} \Lambda_{0,l}(s-;\mathbf{z}_0)]\|_{\infty} = o_{as*}(t)$ as a consequence of Theorem 1, condition C1 and the fact that $\sqrt{n}\mathbb{P}_n\xi = O_p(1)$ by the central limit theorem, lead to the conclusion that $F'''_{n,j} = o_p(1)$. Therefore, $\|\hat{W}^F_{n,j}(t;\mathbf{z}_0) - \tilde{W}^F_{n,j}(t;\mathbf{z}_0)\|_{\infty} = o_p(1)$ for all $j = 1, \ldots, k$ and the proof of the last statement of Theorem 4 is complete.

3 Estimated Influence Functions

In Theorem 2 we provided the consistent estimator of the covariance matrix of the finite-dimensional parameters as

$$\hat{\boldsymbol{\Sigma}}_j = \frac{1}{n} \sum_{i=1}^{n} (\hat{\boldsymbol{\psi}}_{ij} + \hat{\mathbf{R}}_j \hat{\boldsymbol{\omega}}_i)^{\otimes 2}.$$

The first component of the estimated influence functions in $\hat{\boldsymbol{\Sigma}}_j$ is

$$\hat{\boldsymbol{\psi}}_{ij} = \mathbf{H}_{n,j}^{-1}(\hat{\boldsymbol{\beta}}_{n,j}) \int_0^\tau [\mathbf{Z}_i - E_n(t, \hat{\boldsymbol{\beta}}_{n,j})] d\hat{M}_{ij}(t; \hat{\boldsymbol{\beta}}_{n,j}, \hat{\boldsymbol{\gamma}}_n)$$

for $i = 1, \dots, n$ and $j = 1, \dots, k$, where

$$\mathbf{H}_{n,j}(\hat{\boldsymbol{\beta}}_{n,j}) = \frac{1}{n} \int_0^\tau \left\{ \frac{\sum_{l=1}^n \mathbf{Z}_l^{\otimes 2} Y_l(t) e^{\hat{\boldsymbol{\beta}}_{n,j}^T \mathbf{Z}_l}}{\sum_{l=1}^n Y_l(t) e^{\hat{\boldsymbol{\beta}}_{n,j}^T \mathbf{Z}_l}} - \left[\frac{\sum_{l=1}^n Z_l Y_l(t) e^{\hat{\boldsymbol{\beta}}_{n,j}^T \mathbf{Z}_l}}{\sum_{l=1}^n Y_l(t) e^{\hat{\boldsymbol{\beta}}_{n,j}^T \mathbf{Z}_l}} \right]^{\otimes 2} \right\} \sum_{i=1}^n d\tilde{N}_{ij}(t; \hat{\boldsymbol{\gamma}}_n),$$

$$E_n(t, \hat{\boldsymbol{\beta}}_{n,j}) = \frac{\sum_{i=1}^n \mathbf{Z}_i Y_i(t) e^{\hat{\boldsymbol{\beta}}_{n,j}^T \mathbf{Z}_i}}{\sum_{i=1}^n Y_i(t) e^{\hat{\boldsymbol{\beta}}_{n,j}^T \mathbf{Z}_i}}$$

and

$$\hat{M}_{ij}(t; \hat{\boldsymbol{\beta}}_{n,j}, \hat{\boldsymbol{\gamma}}_n) = \tilde{N}_{ij}(t; \hat{\boldsymbol{\gamma}}_n) - \int_0^t Y_i(s) \exp(\hat{\boldsymbol{\beta}}_{n,j}^T \mathbf{Z}_i) d\hat{\Lambda}_{n,j}(s).$$

The second component is

$$\hat{\mathbf{R}}_j = \mathbf{H}_{n,j}^{-1}(\hat{\boldsymbol{\beta}}_{n,j}) \left\{ \frac{1}{n} \sum_{i=1}^n (1 - R_i) \int_0^\tau [\mathbf{Z}_i - E_n(t, \hat{\boldsymbol{\beta}}_{n,j})] dN_i(t) \dot{\pi}_j(\mathbf{W}_i, \hat{\boldsymbol{\gamma}}_n)^T \right\},$$

and $\hat{\boldsymbol{\omega}}_i$ is the usual influence function for the parametric multinomial logit model, where $\boldsymbol{\gamma}_0$ has been replaced by $\hat{\boldsymbol{\gamma}}_n$ and the expectations by sample averages.

The estimated influence function components that are involved in the covariance function estimator for the baseline cumulative cause-specific hazard estimator are

$$\hat{\phi}_{ij}(t) = \int_0^t \frac{d\hat{M}_{ij}(s; \hat{\boldsymbol{\beta}}_{n,j}, \hat{\boldsymbol{\gamma}}_n)}{n^{-1} \sum_{l=1}^n Y_l(s) e^{\hat{\boldsymbol{\beta}}_{n,j}^T \mathbf{Z}_l}} - (\hat{\boldsymbol{\psi}}_{ij} + \hat{R}_j \hat{\boldsymbol{\omega}}_i)^T \int_0^t E_n(s, \hat{\boldsymbol{\beta}}_{n,j}) d\hat{\Lambda}_{n,j}(s)$$

and

$$\hat{\mathbf{R}}_j^\star(t) = \left\{ \frac{1}{n} \sum_{i=1}^n \left[(1 - R_i) \dot{\pi}_j(\mathbf{W}_i, \hat{\boldsymbol{\gamma}}_n) \int_0^t \frac{dN_i(s)}{n^{-1} \sum_{l=1}^n Y_l(s) e^{\hat{\boldsymbol{\beta}}_{n,j}^T \mathbf{Z}_l}} \right] \right\}^T.$$

Finally, the estimated influence functions for the covariance function estimator of the covariate-specific cumulative incidence function estimator are

$$\begin{aligned} \hat{\phi}_{ij}^F(t; \mathbf{z}_0) = & \int_0^t \exp\left[-\sum_{l=1}^k \hat{\Lambda}_{n,l}(s-; \mathbf{z}_0) \right] d\hat{\phi}_{ij}^\Lambda(s; \mathbf{z}_0) \\ & - \int_0^t \left[\sum_{l=1}^k \hat{\phi}_{il}^\Lambda(s-; \mathbf{z}_0) \right] \exp\left[-\sum_{l=1}^k \hat{\Lambda}_{n,l}(s-; \mathbf{z}_0) \right] d\hat{\Lambda}_{n,j}(s; \mathbf{z}_0), \end{aligned}$$

where

$$\hat{\phi}_{ij}^\Lambda(t; \mathbf{z}_0) = [\mathbf{z}_0^T (\hat{\boldsymbol{\psi}}_{ij} + \hat{\mathbf{R}}_j \hat{\boldsymbol{\omega}}_i) \hat{\Lambda}_{n,j}(t) + \hat{\phi}_{ij}(t) + \hat{\mathbf{R}}_j^\star(t) \hat{\boldsymbol{\omega}}_i] \exp(\hat{\boldsymbol{\beta}}_{n,j}^T \mathbf{z}_0).$$

Table 1 Simulation results for β_1 under scenario 3 where the model $\pi_1(\mathbf{W}, \gamma)$ was misspecified with $\eta = 2$.

n	p_m	Method	Bias	MCSD	ASE	CP	MSE	RE
200	23%	Proposed MPPLE	0.008	0.352	0.348	0.953	0.124	1.000
		AIPW	0.010	0.351	0.359	0.954	0.123	0.994
		MI(5)	0.001	0.371	0.360	0.942	0.138	1.108
	40%	Proposed MPPLE	0.010	0.361	0.361	0.951	0.131	1.000
		AIPW	0.013	0.362	0.377	0.962	0.131	1.001
		MI(5)	-0.006	0.391	0.379	0.947	0.153	1.170
	54%	Proposed MPPLE	0.004	0.387	0.378	0.949	0.150	1.000
		AIPW	0.007	0.398	0.411	0.948	0.158	1.054
		MI(5)	-0.011	0.408	0.402	0.951	0.167	1.109
400	23%	Proposed MPPLE	-0.000	0.246	0.247	0.957	0.060	1.000
		AIPW	0.001	0.246	0.248	0.954	0.061	1.006
		MI(5)	-0.010	0.252	0.251	0.950	0.064	1.056
	40%	Proposed MPPLE	-0.002	0.256	0.257	0.957	0.066	1.000
		AIPW	-0.001	0.260	0.260	0.951	0.067	1.025
		MI(5)	-0.012	0.265	0.262	0.950	0.071	1.071
	54%	Proposed MPPLE	0.001	0.272	0.269	0.955	0.074	1.000
		AIPW	0.005	0.279	0.279	0.943	0.078	1.058
		MI(5)	-0.017	0.276	0.275	0.945	0.077	1.034
2000	23%	Proposed MPPLE	-0.001	0.110	0.111	0.945	0.012	1.000
		AIPW	0.001	0.110	0.110	0.943	0.012	1.001
		MI(5)	0.002	0.111	0.110	0.943	0.012	1.019
	40%	Proposed MPPLE	-0.001	0.115	0.115	0.949	0.013	1.000
		AIPW	0.002	0.115	0.115	0.943	0.013	1.007
		MI(5)	0.003	0.117	0.115	0.938	0.014	1.041
	54%	Proposed MPPLE	-0.002	0.119	0.120	0.950	0.014	1.000
		AIPW	0.000	0.120	0.121	0.958	0.015	1.026
		MI(5)	0.004	0.124	0.121	0.939	0.015	1.085

p_m: percent of missingness; MCSD: Monte Carlo standard deviation; ASE: average estimated standard error; CP: coverage probability; MSE: mean squared error; RE: variance of the estimator to variance of the proposed MPPLE (relative efficiency); MPPLE: maximum partial pseudolikelihood estimator; AIPW: augmented inverse probability weighting estimator; MI(5): Lu & Tsiatis type B multiple imputation based on 5 imputations

4 Additional Simulation Results

Simulation results for β_1 under more pronounced misspecification of the model $\pi_1(\mathbf{W}, \gamma)$ are presented in Tables 1-2. Simulation results for the infinite-dimentional parameters are presented in Tables 3-8.

Table 2 Simulation results for β_1 under scenario 4 where the model $\pi_1(\mathbf{W}, \gamma)$ was misspecified with $\eta = 0.1$.

n	p_m	Method	Bias	MCSD	ASE	CP	MSE	RE
200	30%	Proposed MPPLE	0.009	0.492	0.478	0.948	0.242	1.000
		AIPW	0.005	0.492	0.508	0.954	0.242	1.001
		MI(5)	-0.007	0.507	0.514	0.953	0.257	1.062
	49%	Proposed MPPLE	0.011	0.522	0.518	0.949	0.273	1.000
		AIPW	0.006	0.527	0.563	0.962	0.277	1.017
		MI(5)	-0.017	0.560	0.571	0.949	0.314	1.150
	62%	Proposed MPPLE	0.011	0.593	0.563	0.933	0.351	1.000
		AIPW	-0.002	0.605	0.645	0.962	0.366	1.043
		MI(5)	-0.031	0.631	0.639	0.954	0.399	1.133
400	30%	Proposed MPPLE	-0.012	0.354	0.342	0.935	0.126	1.000
		AIPW	-0.015	0.354	0.348	0.938	0.126	0.999
		MI(5)	-0.004	0.358	0.351	0.940	0.128	1.020
	49%	Proposed MPPLE	-0.014	0.390	0.371	0.944	0.153	1.000
		AIPW	-0.022	0.396	0.382	0.937	0.158	1.030
		MI(5)	-0.005	0.394	0.384	0.939	0.155	1.018
	62%	Proposed MPPLE	-0.009	0.429	0.407	0.949	0.184	1.000
		AIPW	-0.018	0.445	0.430	0.941	0.199	1.076
		MI(5)	-0.003	0.433	0.424	0.941	0.188	1.019
2000	30%	Proposed MPPLE	0.008	0.149	0.152	0.949	0.022	1.000
		AIPW	0.008	0.150	0.151	0.945	0.023	1.010
		MI(5)	0.001	0.149	0.153	0.953	0.022	1.004
	49%	Proposed MPPLE	0.006	0.161	0.164	0.949	0.026	1.000
		AIPW	0.005	0.164	0.165	0.953	0.027	1.040
		MI(5)	-0.002	0.164	0.166	0.947	0.027	1.035
	62%	Proposed MPPLE	0.004	0.175	0.180	0.952	0.031	1.000
		AIPW	0.003	0.179	0.183	0.943	0.032	1.054
		MI(5)	-0.004	0.179	0.183	0.955	0.032	1.050

p_m: percent of missingness; MCSD: Monte Carlo standard deviation; ASE: average estimated standard error; CP: coverage probability; MSE: mean squared error; RE: variance of the estimator to variance of the proposed MPPLE (relative efficiency); MPPLE: maximum partial pseudolikelihood estimator; AIPW: augmented inverse probability weighting estimator; MI(5): Lu & Tsiatis type B multiple imputation based on 5 imputations

Table 3 Pointwise simulation results for the proposed MPPLE method in terms of the baseline cumulative cause-specific hazard function $\Lambda_{0,1}(t)$ and the baseline cumulative incidence function $F_{0,1}(t)$, under a correctly specified model $\pi_1(\mathbf{W}, \boldsymbol{\gamma})$ (scenario 1).

			$\Lambda_{0,1}(t)$				$F_{0,1}(t)$			
n	p_m	t	Bias	MCSD	ASE	CP	Bias	MCSD	ASE	CP
200	25%	$\tau_{0.1}$	0.001	0.053	0.053	0.971	0.002	0.041	0.041	0.935
		$\tau_{0.2}$	0.003	0.097	0.096	0.956	0.003	0.057	0.058	0.942
		$\tau_{0.4}$	0.007	0.192	0.184	0.943	0.003	0.071	0.071	0.939
		$\tau_{0.8}$	0.033	0.428	0.397	0.938	0.005	0.074	0.073	0.941
	56%	$\tau_{0.1}$	0.002	0.061	0.059	0.953	0.002	0.047	0.046	0.935
		$\tau_{0.2}$	0.003	0.113	0.108	0.951	0.002	0.067	0.067	0.937
		$\tau_{0.4}$	0.009	0.219	0.206	0.945	0.002	0.085	0.084	0.928
		$\tau_{0.8}$	0.035	0.483	0.440	0.942	0.003	0.090	0.089	0.929
400	25%	$\tau_{0.1}$	0.001	0.038	0.038	0.951	0.001	0.029	0.029	0.946
		$\tau_{0.2}$	0.003	0.070	0.069	0.947	0.001	0.041	0.042	0.950
		$\tau_{0.4}$	0.007	0.135	0.131	0.942	0.000	0.050	0.051	0.941
		$\tau_{0.8}$	0.012	0.293	0.280	0.949	0.002	0.052	0.052	0.943
	56%	$\tau_{0.1}$	0.001	0.042	0.042	0.949	0.000	0.033	0.033	0.951
		$\tau_{0.2}$	0.003	0.078	0.077	0.941	-0.000	0.047	0.048	0.952
		$\tau_{0.4}$	0.007	0.150	0.147	0.950	-0.000	0.059	0.060	0.948
		$\tau_{0.8}$	0.014	0.330	0.310	0.943	0.001	0.062	0.064	0.951
2000	25%	$\tau_{0.1}$	-0.000	0.017	0.017	0.964	-0.000	0.013	0.013	0.956
		$\tau_{0.2}$	-0.001	0.029	0.031	0.950	-0.000	0.018	0.019	0.955
		$\tau_{0.4}$	-0.000	0.056	0.058	0.953	-0.000	0.022	0.023	0.954
		$\tau_{0.8}$	0.000	0.121	0.124	0.959	0.000	0.022	0.023	0.952
	56%	$\tau_{0.1}$	0.000	0.018	0.019	0.956	0.000	0.014	0.015	0.955
		$\tau_{0.2}$	-0.000	0.033	0.034	0.958	-0.000	0.021	0.021	0.954
		$\tau_{0.4}$	-0.001	0.063	0.065	0.955	-0.000	0.026	0.027	0.951
		$\tau_{0.8}$	-0.000	0.134	0.137	0.956	0.000	0.028	0.029	0.948

p_m: percent of missingness; τ_p: p% of the total follow-up time τ; MCSD: Monte Carlo standard deviation of the estimates; ASE: average of standard error estimates; CP: coverage probability

Table 4 Pointwise simulation results for the proposed MPPLE method in terms of the baseline cumulative cause-specific hazard function $\Lambda_{0,1}(t)$ and the baseline cumulative incidence function $F_{0,1}(t)$, under a misspecified model $\pi_1(\mathbf{W}, \boldsymbol{\gamma})$ with $\eta = 0.5$ (scenario 2).

				$\Lambda_{0,1}(t)$				$F_{0,1}(t)$		
n	p_m	t	Bias	MCSD	ASE	CP	Bias	MCSD	ASE	CP
200	27%	$\tau_{0.1}$	0.007	0.056	0.058	0.965	0.009	0.039	0.040	0.946
		$\tau_{0.2}$	-0.001	0.101	0.102	0.958	0.006	0.053	0.055	0.939
		$\tau_{0.4}$	-0.005	0.196	0.193	0.959	0.003	0.066	0.068	0.944
		$\tau_{0.8}$	0.027	0.428	0.412	0.947	0.006	0.071	0.071	0.936
	59%	$\tau_{0.1}$	0.018	0.070	0.069	0.942	0.020	0.051	0.050	0.943
		$\tau_{0.2}$	0.002	0.119	0.117	0.955	0.011	0.067	0.067	0.936
		$\tau_{0.4}$	-0.012	0.225	0.216	0.955	0.004	0.083	0.083	0.929
		$\tau_{0.8}$	0.027	0.489	0.463	0.948	0.006	0.089	0.088	0.936
400	27%	$\tau_{0.1}$	0.010	0.042	0.042	0.950	0.009	0.028	0.029	0.953
		$\tau_{0.2}$	0.003	0.074	0.073	0.939	0.004	0.038	0.039	0.946
		$\tau_{0.4}$	-0.004	0.141	0.138	0.945	0.001	0.047	0.048	0.952
		$\tau_{0.8}$	0.007	0.297	0.289	0.954	0.002	0.050	0.050	0.951
	59%	$\tau_{0.1}$	0.019	0.050	0.049	0.930	0.018	0.034	0.035	0.952
		$\tau_{0.2}$	0.003	0.085	0.084	0.946	0.008	0.046	0.048	0.954
		$\tau_{0.4}$	-0.015	0.157	0.154	0.955	-0.000	0.057	0.059	0.953
		$\tau_{0.8}$	0.006	0.342	0.326	0.951	0.001	0.061	0.063	0.961
2000	27%	$\tau_{0.1}$	0.007	0.018	0.018	0.928	0.008	0.013	0.013	0.914
		$\tau_{0.2}$	-0.002	0.031	0.032	0.961	0.003	0.017	0.018	0.961
		$\tau_{0.4}$	-0.012	0.060	0.061	0.953	0.000	0.021	0.022	0.950
		$\tau_{0.8}$	-0.009	0.124	0.128	0.959	0.001	0.022	0.023	0.960
	59%	$\tau_{0.1}$	0.018	0.022	0.022	0.851	0.019	0.016	0.016	0.795
		$\tau_{0.2}$	-0.000	0.036	0.037	0.962	0.010	0.021	0.021	0.947
		$\tau_{0.4}$	-0.024	0.067	0.068	0.948	0.002	0.026	0.026	0.943
		$\tau_{0.8}$	-0.018	0.140	0.143	0.954	0.002	0.028	0.028	0.945

p_m: percent of missingness; τ_p: p% of the total follow-up time τ; MCSD: Monte Carlo standard deviation of the estimates; ASE: average of standard error estimates; CP: coverage probability

Table 5 Pointwise simulation results for the proposed MPPLE method in terms of the baseline cumulative cause-specific hazard function $\Lambda_{0,1}(t)$ and the baseline cumulative incidence function $F_{0,1}(t)$, under a misspecified model $\pi_1(\mathbf{W}, \boldsymbol{\gamma})$ with $\eta = 2$ (scenario 3).

			$\Lambda_{0,1}(t)$				$F_{0,1}(t)$			
n	p_m	t	Bias	MCSD	ASE	CP	Bias	MCSD	ASE	CP
200	23%	$\tau_{0.1}$	-0.004	0.048	0.049	0.958	-0.002	0.039	0.040	0.928
		$\tau_{0.2}$	-0.004	0.088	0.087	0.954	-0.002	0.056	0.057	0.947
		$\tau_{0.4}$	0.004	0.171	0.165	0.953	-0.000	0.068	0.071	0.945
		$\tau_{0.8}$	0.027	0.357	0.346	0.950	0.003	0.069	0.069	0.937
	54%	$\tau_{0.1}$	-0.009	0.049	0.050	0.960	-0.006	0.041	0.041	0.911
		$\tau_{0.2}$	-0.008	0.092	0.090	0.949	-0.007	0.059	0.060	0.928
		$\tau_{0.4}$	0.008	0.181	0.175	0.953	-0.002	0.074	0.077	0.949
		$\tau_{0.8}$	0.036	0.379	0.365	0.950	0.003	0.078	0.078	0.936
400	23%	$\tau_{0.1}$	-0.003	0.035	0.035	0.951	-0.003	0.028	0.029	0.938
		$\tau_{0.2}$	-0.003	0.063	0.062	0.950	-0.004	0.040	0.041	0.947
		$\tau_{0.4}$	0.006	0.118	0.118	0.940	-0.002	0.050	0.051	0.958
		$\tau_{0.8}$	0.017	0.244	0.244	0.962	0.000	0.049	0.050	0.949
	54%	$\tau_{0.1}$	-0.009	0.035	0.035	0.939	-0.007	0.029	0.029	0.927
		$\tau_{0.2}$	-0.010	0.066	0.064	0.941	-0.009	0.042	0.043	0.944
		$\tau_{0.4}$	0.004	0.127	0.124	0.939	-0.003	0.053	0.055	0.959
		$\tau_{0.8}$	0.024	0.265	0.258	0.954	0.001	0.054	0.055	0.947
2000	23%	$\tau_{0.1}$	-0.004	0.016	0.016	0.944	-0.004	0.013	0.013	0.936
		$\tau_{0.2}$	-0.004	0.026	0.028	0.960	-0.004	0.017	0.018	0.953
		$\tau_{0.4}$	0.001	0.051	0.052	0.949	-0.001	0.022	0.023	0.950
		$\tau_{0.8}$	0.004	0.107	0.108	0.956	-0.000	0.022	0.022	0.949
	54%	$\tau_{0.1}$	-0.009	0.016	0.016	0.918	-0.008	0.013	0.013	0.897
		$\tau_{0.2}$	-0.009	0.028	0.029	0.946	-0.008	0.018	0.019	0.933
		$\tau_{0.4}$	0.002	0.055	0.055	0.949	-0.003	0.024	0.025	0.955
		$\tau_{0.8}$	0.012	0.113	0.114	0.952	-0.000	0.025	0.025	0.947

p_m: percent of missingness; τ_p: p% of the total follow-up time τ; MCSD: Monte Carlo standard deviation of the estimates; ASE: average of standard error estimates; CP: coverage probability

Table 6 Pointwise simulation results for the proposed MPPLE method in terms of the baseline cumulative cause-specific hazard function $\Lambda_{0,1}(t)$ and the baseline cumulative incidence function $F_{0,1}(t)$, under a misspecified model $\pi_1(\mathbf{W}, \boldsymbol{\gamma})$ with $\eta = 0.1$ (scenario 4).

				$\Lambda_{0,1}(t)$				$F_{0,1}(t)$		
n	p_m	t	Bias	MCSD	ASE	CP	Bias	MCSD	ASE	CP
200	30%	$\tau_{0.1}$	-0.003	0.069	0.067	0.953	0.008	0.032	0.031	0.937
		$\tau_{0.2}$	-0.015	0.117	0.116	0.952	0.004	0.043	0.042	0.932
		$\tau_{0.4}$	-0.014	0.229	0.222	0.941	0.004	0.055	0.054	0.932
		$\tau_{0.8}$	0.031	0.498	0.466	0.942	0.006	0.060	0.060	0.942
	62%	$\tau_{0.1}$	-0.003	0.082	0.078	0.941	0.016	0.045	0.041	0.936
		$\tau_{0.2}$	-0.030	0.133	0.127	0.937	0.005	0.054	0.052	0.921
		$\tau_{0.4}$	-0.032	0.256	0.242	0.935	0.001	0.067	0.065	0.934
		$\tau_{0.8}$	0.029	0.559	0.518	0.945	0.004	0.072	0.071	0.936
400	30%	$\tau_{0.1}$	0.001	0.050	0.048	0.949	0.008	0.022	0.022	0.958
		$\tau_{0.2}$	-0.012	0.086	0.083	0.940	0.002	0.030	0.030	0.951
		$\tau_{0.4}$	-0.008	0.168	0.160	0.943	0.002	0.039	0.039	0.943
		$\tau_{0.8}$	0.014	0.340	0.329	0.942	0.003	0.042	0.042	0.948
	62%	$\tau_{0.1}$	-0.001	0.059	0.057	0.945	0.017	0.030	0.030	0.952
		$\tau_{0.2}$	-0.035	0.096	0.091	0.920	0.004	0.037	0.038	0.956
		$\tau_{0.4}$	-0.040	0.187	0.175	0.931	0.001	0.046	0.047	0.947
		$\tau_{0.8}$	-0.005	0.387	0.367	0.946	0.002	0.050	0.051	0.944
2000	30%	$\tau_{0.1}$	-0.003	0.022	0.021	0.943	0.008	0.010	0.010	0.893
		$\tau_{0.2}$	-0.023	0.036	0.036	0.908	0.001	0.014	0.013	0.938
		$\tau_{0.4}$	-0.025	0.068	0.070	0.936	0.000	0.017	0.017	0.942
		$\tau_{0.8}$	-0.018	0.138	0.144	0.954	0.001	0.019	0.019	0.947
	62%	$\tau_{0.1}$	-0.000	0.026	0.025	0.938	0.020	0.014	0.014	0.746
		$\tau_{0.2}$	-0.042	0.039	0.040	0.823	0.006	0.017	0.017	0.939
		$\tau_{0.4}$	-0.055	0.074	0.077	0.907	0.002	0.021	0.021	0.950
		$\tau_{0.8}$	-0.033	0.155	0.160	0.948	0.003	0.023	0.023	0.947

p_m: percent of missingness; τ_p: p% of the total follow-up time τ; MCSD: Monte Carlo standard deviation of the estimates; ASE: average of standard error estimates; CP: coverage probability

Table 7 Simulation results for the coverage probability of the proposed 95% simultaneous confidence bands for $\Lambda_{0,1}(t)$ and $F_{0,1}(t)$ under a correctly specified model $\pi_1(\mathbf{W}, \boldsymbol{\gamma})$ (scenario 1).

		$\Lambda_{0,1}(t)$		$F_{0,1}(t)$	
n	p_m	EP	HW	EP	HW
200	25%	0.947	0.953	0.944	0.952
	44%	0.949	0.949	0.946	0.948
	56%	0.942	0.949	0.941	0.949
400	25%	0.944	0.944	0.948	0.943
	44%	0.945	0.945	0.950	0.952
	56%	0.945	0.940	0.954	0.950
2000	25%	0.950	0.950	0.952	0.959
	44%	0.956	0.959	0.956	0.956
	56%	0.956	0.958	0.948	0.954

$\bar{p}_m$: average percent of missingness over the two scenarios; EP: equal-precision-type band; HW: Hall–Wellner-type band

Table 8 Simulation results for the coverage probability of the proposed 95% simultaneous confidence bands for $\Lambda_{0,1}(t)$ and $F_{0,1}(t)$ under misspecified models $\pi_1(\mathbf{W},\gamma)$ (scenarios 2-4).

				$\Lambda_{0,1}(t)$		$F_{0,1}(t)$	
Scenario	η	n	p_m	EP	HW	EP	HW
2	0.5	200	27%	0.901	0.924	0.922	0.950
			46%	0.827	0.887	0.867	0.924
			59%	0.757	0.835	0.810	0.898
		400	27%	0.867	0.900	0.912	0.941
			46%	0.757	0.829	0.809	0.919
			59%	0.656	0.754	0.728	0.886
		2000	27%	0.693	0.855	0.772	0.905
			46%	0.298	0.586	0.387	0.746
			59%	0.121	0.325	0.163	0.508
3	2	200	23%	0.946	0.945	0.947	0.953
			40%	0.960	0.954	0.947	0.949
			54%	0.956	0.957	0.941	0.950
		400	23%	0.948	0.946	0.946	0.955
			40%	0.947	0.950	0.942	0.955
			54%	0.944	0.941	0.934	0.952
		2000	23%	0.939	0.958	0.938	0.951
			40%	0.936	0.946	0.928	0.938
			54%	0.927	0.938	0.914	0.933
4	0.1	200	30%	0.682	0.781	0.854	0.921
			49%	0.431	0.524	0.646	0.792
			62%	0.271	0.326	0.476	0.618
		400	30%	0.601	0.718	0.829	0.924
			49%	0.203	0.296	0.514	0.708
			62%	0.083	0.123	0.284	0.420
		2000	30%	0.160	0.325	0.617	0.836
			49%	0.000	0.003	0.080	0.220
			62%	0.000	0.000	0.005	0.011

$\bar{p}_m$: average percent of missingness over the two scenarios; EP: equal-precision-type band; HW: Hall–Wellner-type band

5 Results from the Analysis of the Bladder Cancer Trial Data

This section includes the results from the application of the proposed methods to the EORTC bladder cancer trial data.

Table 9 Descriptive statistics for the EORTC bladder cancer trial.

	BCG treatment group	
	1/3 dose<br>n (%)	Full dose<br>n (%)
Vital status		
Alive	258 (75.7)	251 (74.0)
Deceased	83 (24.3)	88 (26.0)
Cause of death		
Bladder cancer	13 (15.7)	20 (22.7)
Other causes	55 (66.3)	60 (68.2)
Missing	15 (18.1)	8 (9.1)
WHO performance status		
Fully active	281 (82.4)	278 (82.0)
Reduced activity	60 (17.6)	61 (18.0)
	Median (IQR)	Median (IQR)
Age (years)	68.0 (60.0, 74.0)	67.0 (58.0, 73.0)

WHO: World Health Organization

Table 10 Data analysis of the EORTC bladder cancer trial sample.

	Proposed MPPLE			AIPW		
Covariate	$\hat{\beta}_n$	SE	p-value	$\hat{\beta}_n$	SE	p-value
Death due to bladder cancer						
Group (Full dose = 1, 1/3 dose = 0)	0.451	0.356	0.204	0.421	0.372	0.258
Age (years)	0.026	0.019	0.174	0.027	0.021	0.191
Fully active (no = 1, yes = 0)	0.488	0.412	0.237	0.512	0.453	0.259
Death from other causes						
Group (Full dose = 1, 1/3 dose = 0)	0.084	0.175	0.633	0.091	0.184	0.620
Age (years)	0.085	0.013	<0.001	0.085	0.013	<0.001
Fully active (no = 1, yes = 0)	0.105	0.221	0.635	0.097	0.214	0.650

MPPLE: maximum pseudo partial likelihood estimator; AIPW: augmented inverse probability weighting estimator; SE: estimated standard error of $\hat{\beta}_n$

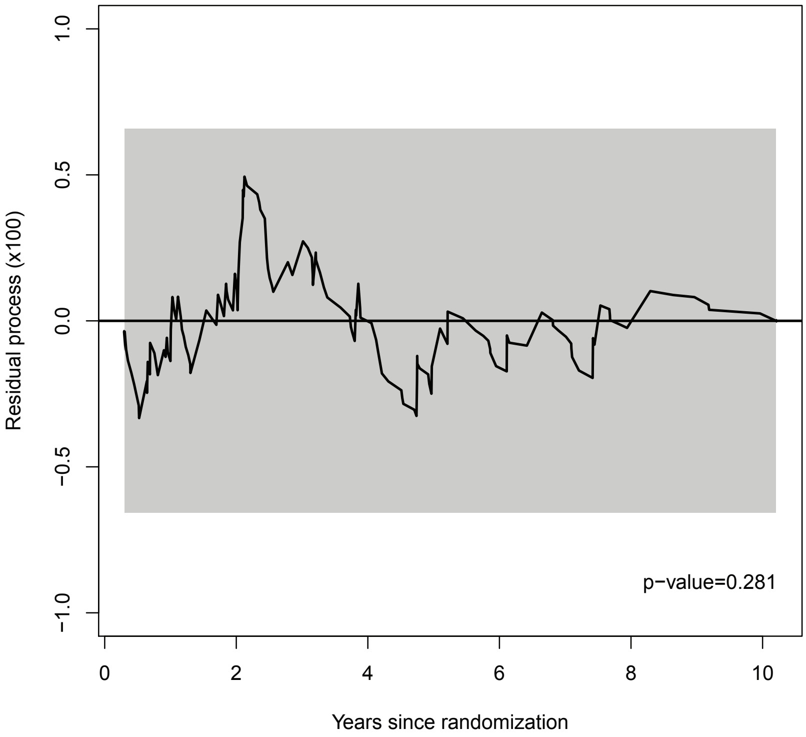

Fig. 1 Cumulative residual process for the evaluation of the parametric model $\pi_1(\boldsymbol{W}, \gamma_0)$ based on the EORTC bladder cancer trial data along with the 95% goodness-of-fit band (grey area) and the corresponding p-value.

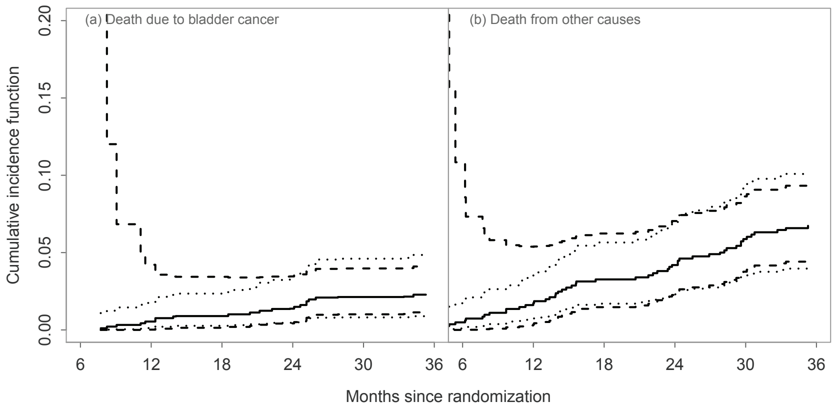

Fig. 2 Predicted cumulative incidence functions (solid lines) of death from (a) bladder cancerr and (b) other causes, for a 68-year old patient who is fully active and who was assigned in the 1/3 dose of BCG, along with the 95% simultaneous confidence bands based on equal precision (dotted lines) and Hall–Wellner-type weights (dashed lines). The time interval depicted was restricted according to the guidlines provided in subsection 3.2 of the main text.

References

1. Cheng, S.C., Fine, J.P., Wei, L.J.: Prediction of cumulative incidence function under the proportional hazards model. Biometrics **54**, 219–228 (1998)
2. Kosorok, M.R.: Introduction to Empirical Processes and Semiparametric Inference. Springer, New York (2008)
3. Spiekerman, C.F., Lin, D.Y.: Marginal regression models for multivariate failure time data. Journal of the American Statististical Association **93**, 1164–1175 (1998)
4. van der Vaart, A.W., Wellner, J.A.: Weak Convergence and Empirical Processes with Applications to Statistics. Springer-Verlag, New York (1996)
